# Complete Genome Sequence of *Enterobacter cloacae* UW5, a Rhizobacterium Capable of High Levels of Indole-3-Acetic Acid Production

**DOI:** 10.1128/genomeA.00843-15

**Published:** 2015-08-06

**Authors:** Thomas J. D. Coulson, Cheryl L. Patten

**Affiliations:** Department of Biology, University of New Brunswick, Fredericton, New Brunswick, Canada

## Abstract

We report the complete genome sequence of *Enterobacter cloacae* UW5, an indole-3-acetic acid-producing rhizobacterium originally isolated from the rhizosphere of grass. The 4.9-Mbp genome has a G+C content of 54% and contains 4,496 protein-coding sequences.

## GENOME ANNOUNCEMENT

Members of the *Enterobacter* genus are Gram-negative facultative anaerobic bacteria commonly found inhabiting the rhizosphere of plants, and they may also be members of the intestinal microflora of humans. *Enterobacter cloacae* strains are often endophytes capable of biocontrol of plant infectious diseases or production of plant growth-stimulating compounds (1). *E. cloacae* UW5 was originally isolated from the rhizosphere of reeds in Waterloo, Ontario, Canada (2, 3), and has been shown to produce high levels of indole-3-acetic acid (IAA) (4), a signaling molecule that acts as a bacterial stress response regulator, antimicrobial metabolite, and plant growth stimulant (5). Regulation of IAA by the transcription factor TyrR has been characterized in this strain (4, 6). The genome of *E. cloacae* UW5 was sequenced to facilitate further insight into the TyrR regulon and the function of IAA in bacterial physiology and bacterial-plant interactions.

Genomic DNA was extracted from an overnight culture grown at 30°C, using the Wizard Genomic DNA purification kit (Promega). A 100-bp paired-end TruSeq library was prepared with an average fragment size of 444 bp and sequenced in a single Illumina HiSeq 2500 lane (McGill University and Génome Québec Innovation Center, Montreal, Canada). A total of 251,715,967 reads were generated, totaling over 51 billion basses. Seqtk (https://github.com/lh3/seqtk) was used to randomly extract a subset of 10 million forward and reverse paired reads, which were then trimmed using Trimmomatic (7) to remove adapters and low-quality sequences. The resulting reads were assessed for sequence quality using FastQC version 0.11.2 (8) before assembly. Genome assembly was performed using a combination of *de novo* assembly and reference-guided read mapping to a closely related reference genome. An optimal *k*-mer of 71 was chosen using VelvetOptimiser, which was used for *de novo* assembly with Velvet version 1.2.10 (9). A total of 83 contigs were generated, with an average of 330-fold coverage and an *N*_50_ length of 2,836,112 bp. The same read data were then mapped to the *E. cloacae* EcWSU1 reference genome (NC_016514) (10) using CLC Genomics Workbench version 8.0.1 (Qiagen) at both low (80% identity and 50% length fraction) and high (90% identity and 90% length fraction) specificity, and the consensus sequences extracted to produce the reference-guided contigs. Contigs from both *de novo* and reference-guided assemblies were then joined using the CLC Microbial Genome Finishing module to merge overlapping sequences into a single contig. Genome annotation was performed using the NCBI Prokaryotic Genome Annotation Pipeline (PGAP) (http://www.ncbi.nlm.nih.gov/genome/annotation_prok).

The *E. cloacae* UW5 genome consists of a single chromosome that is 4,904,981 bp in length with a G+C content of 54.46%. The genome contains 4,496 genes assigned with protein-encoding function, 77 tRNA genes, and 25 rRNA genes organized into eight rRNA operons.

### Nucleotide sequence accession number.

The complete *E. cloacae* UW5 genome sequence has been deposited in GenBank under the accession number CP011798. The version reported in this paper is the first version.
